# Causal association between thyroid function and the risk of infertility: a Mendelian randomization study

**DOI:** 10.3389/fendo.2024.1425639

**Published:** 2024-10-04

**Authors:** Qinyu Liu, Yingkun Qiu, Jialin Jiang, Shushu Long, Chengyu Zhu, Gang Chen, Junping Wen

**Affiliations:** ^1^ Department of Endocrinology, Key Laboratory of Endocrinology, Shengli Clinical Medical College of Fujian Medical University, Fujian Provincial Hospital, Fuzhou University Affiliated Provincial Hospital, Fuzhou, China; ^2^ Department of Laboratory Medicine, Fujian Medical University Union Hospital, Fuzhou, China

**Keywords:** thyroid dysfunction, thyroid-stimulating hormone, free tetraiodothyronine, infertility, Mendelian randomization

## Abstract

**Objectives:**

Thyroid dysfunction is commonly associated with the risk of infertility in both females and males. However, recent randomized controlled trials have demonstrated that thyroid function levels in females are not significantly related to infertility, and evidence on the association between male thyroid function and infertility is limited. We aim to investigate the association between thyroid function levels and infertility in both females and males.

**Method:**

A two-sample Mendelian randomization study was conducted using four methods, with the inverse variance weighted method (IVW) as the primary approach. Data on thyroid function as the exposure were obtained from the ThyroidOmics Consortium and UK Biobank, including over 700,000 individuals from a large meta-analysis of genome-wide association studies for thyroid function and dysfunction. The outcome data for infertility in both sex encompassed more than 70,000 individuals from the FinnGen Consortium. All participants were adults of European ancestry. The MR Egger regression intercept and Cochran’s Q test were employed to evaluate directional pleiotropy and heterogeneity.

**Results:**

The results indicated no causal effect of thyroid-stimulating hormone (TSH) and free tetraiodothyronine (fT4) on female and male infertility. Furthermore, no causal association between hypo- and hyperthyroidism and infertility were identified. Notably, we observed a causal relationship between high TSH and endometriosis-related infertility (OR=0.82, 95% CI: 0.74–0.91, *P* = 1.49E-04).

**Conclusions:**

This study did not find evidence for casual relationship between thyroid function levels and risk of infertility. The findings suggest that overall thyroid function levels may not be a significant predictor of infertility risk.

## 1 Introduction

Numerous observational studies have established a link between thyroid dysfunction and infertility (1). However, the extent of thyroid dysfunction’s impact on the female reproductive system varies, and its effects on fertility are inconsistent (2–4). The influence of thyroid dysfunction on male fertility remains understudied, despite evidence suggesting a potential connection. Infertility, characterized by the inability to conceive after 12 months of regular, unprotected sexual intercourse, is a significant public health issue with a global prevalence of 8-12% (5–8). Optimal thyroid function is crucial for maintaining reproductive fertility, as thyroid hormones (THs) interact with nuclear receptors in reproductive organs, modulating their development and function (9–11). Changes in sex steroid levels and sex hormone-binding globulin among individuals with hyper- and hypothyroidism regardless of sex has been reported previously (12–16).

In women, infertility is prevalent in those with Hashimoto’s and Graves’ diseases, affecting approximately 50% of patients (17). Elevated thyroid-stimulating hormone (TSH) levels have been observed in women with unexplained infertility compared to controls (18). However, the clinical significance of TSH levels in the high-normal range (2.5–4.9 mIU/L) for pregnancy outcomes has been debated, with some studies showing no adverse effects on clinical pregnancy, live birth, and miscarriage rates (19). A comparison between patients with TSH levels <2.5 mIU/L and those with TSH levels >2.5 mIU/L showed no significant effect on conception, clinical pregnancy, first-trimester pregnancy loss, or live birth rates (20). Furthermore, The role of levothyroxine (LT4) treatment in infertility has also been inconclusive in randomized controlled trials (RCTs) (3, 4).

While the effects of thyroid dysfunction on female reproductive function have been extensively studied, its impact on male reproductive function is less clear. Some studies suggest that thyroid hormone disorders can lead to sexual dysfunction, potentially reversible with normalization of TH levels (21–23). THs are involved in ejaculation physiology and can affect testicular function through nuclear receptors (10, 24–27). However, the evidence linking thyroid dysfunction directly to male infertility is limited, and there is no consensus on the impact of thyroid function on fertility in both sexes.

Autoimmune thyroid diseases significantly impair fertility by creating a cytotoxic environment that negatively affects oocyte maturation and embryonic development. These conditions are particularly prevalent in women with infertility related to endometriosis or polycystic ovary syndrome (PCOS) (28, 29). Despite debates on TSH level benchmarks for thyroid dysfunction, the ATA and ETA propose that women with infertility starting ART take a low-dose levothyroxine regimen to keep TSH under 2.5 mIU/L, four weeks before ovarian stimulation, to enhance fertility treatment outcomes (30, 31).

Given the potential biases in previous observational studies due to limitations and confounding factors, there is a clear need for more robust experimental designs. This study aims to provide clarity by investigating the genetic causal associations between thyroid function, hypothyroidism, hyperthyroidism, and infertility using Mendelian randomization (MR) methodology.

## 2 Methods

### 2.1 Data source

The Genome Wide Association Studies (GWAS) meta-analysis summary statistics pertaining to thyroid function were obtained from the ThyroidOmics Consortium (www.thyroidomics.com), which investigates the determinants and effects of thyroid diseases and thyroid function, using diverse omics technologies in multiple large and well-phenotyped cohort studies. Thyroid function was assessed using sex-stratified TSH and free tetraiodothyronine (fT4) levels as conducted by Teumer et al. (32), whereas sex-stratified statistics for hypo- and hyperthyroidism as binary exposures were obtained from the UK Biobank. To address the potential limitations of clinical diagnosis of hypothyroidism and hyperthyroidism in the outcome data, we included non-sex-stratified high and low TSH traits (compared to cohort-specific reference ranges) as the exposure groups. The numerical values of reference ranges, determined by the 2.5th and 97.5th percentiles of TSH while considering assay variability, demographic traits, and environmental factors such as iodine intake, are similar among the studies included. This case-control GWAS encompassed a total of 27 studies for high TSH and 20 studies for low TSH for subsequent analysis (**Supplementary Table S1**) (33).

The factors that contribute to infertility exhibit substantial differences between men and women, influenced by distinct anatomical structures and physiological processes. This group comprises individuals diagnosed with infertility according to the International Classification of Diseases, Tenth Revision (ICD-10, 2016 version), as reported by the FinnGen Consortium. The FinnGen data was collected through an extensive recruitment process that included hospital biobanks and disease-specific ‘legacy’ cohorts. Our approach to case identification was methodically stratified by sex, ensuring that the outcomes of our MR study accurately capture the distinct impacts of sex on infertility. The case group was identified using FinnGen’s clinical endpoints, which are based on a thorough set of criteria including diagnoses from healthcare registers, hospital discharge summaries, and other relevant medical records (**Supplementary Table S2**). To ensure a valid comparison, the control group was composed of individuals from the general population without the condition of interest. These controls were precisely matched to cases based on key demographic variables—age, sex, and ethnicity—to minimize potential confounding effects. This rigorous matching process is central to the robustness of our study design.

To minimize ancestry-related potential confounding effects, we restricted the analyses to unrelated participants with European ancestry. Significant crossover or overlap was not observed between participants in the GWAS for thyroid function and infertility. Ethical approval was not required since publicly available summary-level statistics were utilized in this study.

### 2.2 Instrumental variable selection criteria

MR necessitates that the genetic instruments exhibit a strong association with thyroid function-related traits (p < 5 × 10^−8^). To account for linkage disequilibrium (r^2^ >0.001), a pruning step was performed on the candidate IVs, ensuring that any association between instruments and the outcome was exposure-driven. Additionally, variants within a 1-Mb distance from other IVs displaying a stronger association were excluded to mitigate bias. These single nucleotide polymorphisms (SNPs) were chosen as IVs. We evaluated the potential weak instrumental bias using the F-statistic, calculated as F= β ^2^_exposure_/SE ^2^_exposure_. SNPs with *F*-statistic <10 was considered weak IVs and excluded from further analysis.

### 2.3 Statistical analysis

Statistical analyses were mainly performed using R software version 4.3.1 (https://www.rproject.org/, accessed on 20 February 2024) with the “Two-Sample MR” package serving as the primary analysis tool. The effect allele was defined as the allele associated with increased TSH and fT4 levels and increased risk of hypothyroidism, hyperthyroidism, and high and low TSH (exposure). The traits of low and high TSH versus reference range TSH may overlap with other exposure factors selected in this study, particularly hypothyroidism and hyperthyroidism. However, addressing the subclinical forms of hyperthyroidism and hypothyroidism cannot be overlooked. The primary analysis involved the inverse variance weighting (IVW) method, which required validating all SNPs as IVs. The weighted median method provided reliable estimates when at least 50% of the weight was derived from valid genetic variants (34). MR Egger yields estimate robust to pleiotropy, even when fewer than 50% of the genetic variants are considered valid (35). The weighted mode method was also applied (36). A *p-*value below 0.05 was considered statistically significant.

Sensitivity analysis was conducted using three methods: heterogeneity testing, horizontal pleiotropy testing, and leave-one-out analysis. Cochran’s Q statistic was used to examine the heterogeneity of individual SNPs in IVW tests, and in cases where heterogeneity was detected (Q*_pval_* < 0.05), the random effects IVW approach was employed. The MR-Egger intercept was used to assess horizontal pleiotropy, and obtaining a significant result in this test is considered unacceptable. The leave-one-out approach systematically eliminates each SNP to assess the individual influence of each SNP.

## 3 Results

For women, we identified 20 SNPs associated with TSH, 11 SNPs associated with fT4, and 13 and 4 SNPs associated with hypothyroidism and hyperthyroidism, respectively. For men, we identified 25 SNPs associated with TSH, 9 SNPs associated with fT4, and 8 and 3 SNPs associated with hypo- and hyperthyroidism, respectively. Moreover, 9 and 14 SNPs associated with high and low TSH (overall), respectively, were selected (**Supplementary Table S3**). The F-statistic values of each SNP at every filtering step were all > 10 (range: 30.05-474.85), indicating that all selected IVs were valid and not susceptible to weak instrument bias. The MR analytical results of association of TSH, fT4, hyperthyroidism, hypothyroidism, and high and low TSH with male and female infertility are presented in **Supplementary Table S4**.

### 3.1 TSH and infertility

No association between one standard deviation (SD) increase in genetically predicted TSH and the risk of infertility in both sexes was observed in the MR analyses (women, IVW: odds ratio [OR]=0.98, 95% confidence interval [CI]: 0.90–1.07, *P* = 0.66; men, IVW: OR=1.06, 95% CI: 0.86–1.30, *P* = 0.59). No evidence of heterogeneity or horizontal pleiotropy was found in the sensitivity analyses (**Supplementary Table S4**; **Supplementary Figures S1**, **S2**).

### 3.2 fT4 and infertility

One SD increase in genetically predicted fT4 was not associated with the risk of female and male infertility after MR analyses (women, IVW: OR=0.99, 95% CI: 0.86–1.14, *P* = 0.89; men, IVW: OR=0.86, 95% CI: 0.51–1.43, *P* = 0.56). Sensitivity analyses revealed heterogeneity (women Q*_pval_* =0.03, men Q*_pval_* =0.01), indicating variability within the study population. However, no evidence of horizontal pleiotropy was observed. Hence, the IVW analysis was conducted using a random effects model (**Supplementary Table S4**; **Supplementary Figures S1**, **S2**).

### 3.3 Hypothyroidism, hyperthyroidism, and infertility

Hypothyroidism was not significantly associated with the risk of female and male infertility (**Supplementary Table S3**). Similarly, the MR analysis did not reveal a causal relationship between hyperthyroidism and infertility in both sexes. No evidence of heterogeneity or horizontal pleiotropy was found after sensitivity analyses (**Supplementary Table S3**). When conducting the analysis on hyperthyroidism, we observed heterogeneity between hyperthyroidism in women and infertility (Q*_pval_* =0.04). Therefore, a random-effects model, IVW, was used to analyze the causal relationship in women. No evidence of horizontal pleiotropy was observed. The results revealed no causal relationship between hyperthyroidism and infertility in both sexes (**Supplementary Table S4**; **Supplementary Figures S1**, **S2**).

### 3.4 High TSH, low TSH, and infertility

High TSH was not significantly associated with the risk of female and male infertility (women, IVW: OR=0.98, 95% CI: 0.92–1.03, *P* = 0.40; men, IVW: OR=1.06, 95% CI: 0.88–1.27, *P* = 0.54). Similarly, the MR analysis did not reveal a causal relationship between low TSH and infertility in both sexes. No evidence of heterogeneity or horizontal pleiotropy was found after the sensitivity analyses (**Supplementary Table S4**; **Supplementary Figures S1**, **S2**).

### 3.5 Thyroid function and infertility caused by various factors

**Figure 1:**
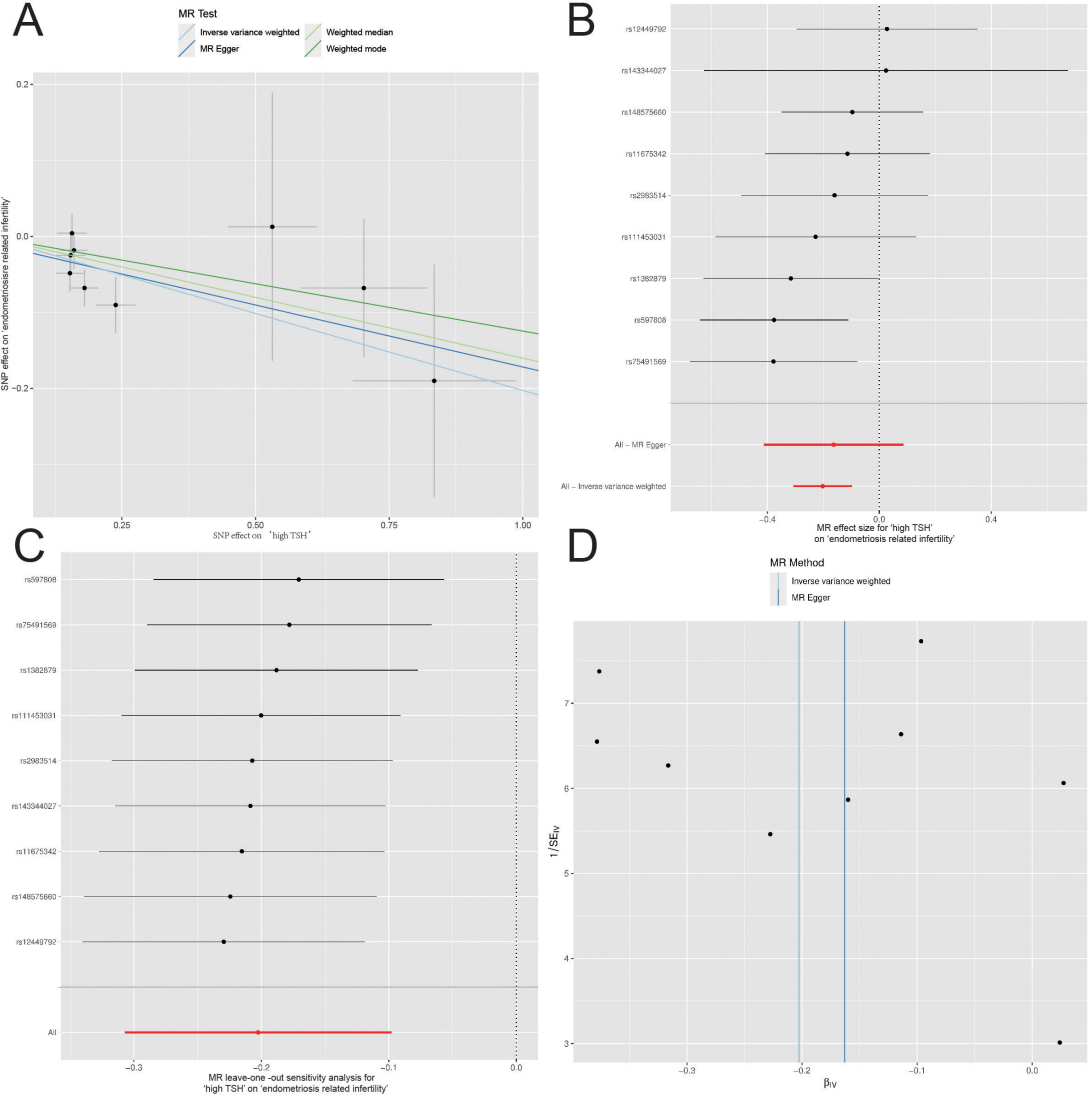
Mendelian Randomization (MR) Analyses Regarding the Effect of High TSH and Endometriosis-related Infertility in Women. **(A)** Scatter plot for instrument high TSH associations against instrument endometriosis-related infertility associations (x-axis). **(B)** Forest plot for the assessment of individual and overall effects of each instrumental variable in the analysis. **(C)** Leave-one-out analysis. **(D)** Funnel plot for visually assess the presence of publication bias and asymmetry in the distribution of estimated causal effects.

The six categories of thyroid function variables (TSH, fT4, hypothyroidism, hyperthyroidism, high TSH, and low TSH) were subjected to MR analysis with five different infertility factors (endometriosis related infertility, cervical and vaginal origin, uterine origin, tubal origin, and anovulation-associated infertility). Notably, a causal association was observed between high TSH and endometriosis-related infertility in women (OR=0.82, 95% CI=0.74–0.91, *P* = 1.49E-04) (**Figure 1**). However, no significant causal associations were observed for other conditions (**Supplementary Table S4**; **Supplementary Figures S3**–**S7**).

## 4 Discussion

This study represents the first comprehensive investigation of the causal relationship between thyroid function and infertility, leveraging genetic variation and a diverse array of MR methods. The implementation of sex-stratified cohorts was pivotal, effectively controlling for confounders and bolstering the internal and external validity of our findings. Our MR analysis, while not establishing a causal nexus between genetically elevated TSH and fT4 levels and infertility, provides novel insights into the complex dynamics between thyroid function and reproductive health. This finding prompts a critical reassessment of the existing body of evidence, highlighting the nuanced relationship between thyroid hormones and fertility.

Our results revealed that genetically increased TSH and fT4 levels in both men and women were not causally associated with infertility. Furthermore, no evidence was found supporting a causal relationship between genetically predicted hypothyroidism, hyperthyroidism, high TSH, low TSH, and infertility in both sexes. Additionally, while we observed a causal relationship between hypothyroidism and endometriosis-related infertility in women, no causal associations were observed between other thyroid function-related exposures and infertility factors. This observation merits further investigation and discussion as it may provide insights into specific subpopulations within the context of infertility.

Previous studies have reported that thyroid function levels may affect fertility in both sexes. Physiological research indicated that hypo- and hyperthyroidism were linked to sex hormone-binding globulin, sex hormone levels, ovarian reserve, and even sexual function (37–44). Multiple studies support the association of male hypothyroidism with abnormal sperm morphology and hyperthyroidism with abnormal sperm motility and DNA damage (37, 45, 46). These research findings suggest that thyroid dysfunction may lead to infertility. However, clinical research on the relationship between male thyroid disorders and infertility is limited. Conversely, for women, numerous observational studies suggest a potential association between thyroid dysfunction and female infertility, with most focusing on hypothyroidism. Observational studies have found that, after excluding absolute causes of infertility, TSH levels exceeding 4.0 mIU/L are closely associated with adverse fertility outcomes, whereas elevated thyroid antibodies are not related to these outcomes (47, 48). Two observational studies targeting subclinical hypothyroidism and infertility populations suggest a potential association between the two (49, 50). Additionally, an association has been observed between elevated TSH levels and reduced clinical pregnancy rates (51). Two RCTs demonstrated that LT4 supplementation in infertile women with subclinical hypothyroidism significantly improved embryo quality, increased clinical pregnancy rates following assisted reproductive technology, and reduced the risk of miscarriage (52, 53).

However, numerous studies have found no evidence of such an association, suggesting that this correlation might be influenced by other endocrine disruptions related to thyroid dysfunction. Despite the heightened risk of miscarriage, cases of spontaneous conception have been observed in individuals with overt hypothyroidism (54, 55). A study found that women with clinical hypothyroidism and subclinical hypothyroidism did not show significant changes in ovarian reserves (56). Moreover, a recent RCT found no association between TSH levels ≥2.5 or the presence of anti-thyroid antibodies and fecundity, pregnancy loss, or live birth in healthy reproductive-aged women with a history of miscarriage (2). Variations in TSH levels, whether within the normal range or reaching subclinical hypothyroidism status, are not associated with clinical pregnancy and delivery rates (57, 58). Additional high-quality RCTs have demonstrated the lack of LT4 supplementation effect on the pregnancy rate among women with subclinical hypothyroidism undergoing *in vitro* fertilization or among women with normal thyroid function but positive for anti-thyroperoxidase antibodies (3, 4, 59, 60). The effect of hyperthyroidism on female fertility remains unclear owing to a lack of reliable evidence (47, 61). Untreated hyperthyroidism in women is associated with a higher risk of spontaneous miscarriage, although most women with hyperthyroidism still experience ovulation (61, 62).

Overall, a cornerstone of our research is the support and expansion of previous observational studies linking thyroid dysfunction with female infertility, particularly hypothyroidism. It is imperative to consider issues such as low quality of evidence and significant heterogeneity in these observational studies, which may arise from small sample sizes, suboptimal study designs, varied selection criteria for infertility, and differences in methods used to measure thyroid autoimmunity, THs, and reference values for TSH. We serendipitously discovered a correlation between high TSH levels and a decreased risk of endometriosis-related infertility, which aligns with a previous study (63). In the balance of TSH and THs, T3 and T4 specifically stimulate the proliferation of ectopic endometrial cells and the production of reactive oxygen species (63, 64). Furthermore, using a mouse model of toxic hypothyroidism, a significant reduction in induced endometriosis through uterine horn surgical implantation was observed (63).

MR analysis offers significant advantages in controlling unmeasured confounding and reverse causation. This is particularly pertinent in studying the relationship between thyroid function and infertility, as thyroid dysfunction often triggers systemic metabolic instability. Our study focused on genetically increased TSH and fT4 levels as primary factors, along with genetic predisposition to hypothyroidism and hyperthyroidism as important supplementary factors, providing a comprehensive evaluation of thyroid function. Notably, we stratified the analysis by sex in the MR study, recognizing significant disparities in thyroid dysfunction prevalence and underlying physiological mechanisms (65). Failure to account for sex differences in the study design could introduce potential limitations in rigor. Our findings not only support numerous observational studies on the association between thyroid dysfunction and female infertility but also contribute significantly to the limited research evidence on the relationship between male thyroid dysfunction and infertility.

However, we acknowledge the limitations inherent in our study design. The utilization of public GWAS databases, while advantageous for sample size, restricted access to granular clinical details. The absence of age stratification and detailed classification of infertility causes, particularly for anovulation-related infertility, limits the depth of our analysis. Additionally, the study’s focus on individuals of European ancestry may impact the broader generalizability of our findings.

Although a causal relationship between high TSH levels and a decreased risk of endometriosis-related infertility was found, no association was observed with other thyroid functions as potential exposures due to the negative feedback regulation between TSH and THs. Additionally, the study did not explore reverse causality because of insufficient instrumental variables for sex-stratified infertility. Additionally, the distribution of female infertility causes should be interpreted within the context of the Finnish population, as the stratified data on female infertility included may not accurately reflect the actual epidemiology (6). In light of these considerations, future research should endeavor to employ larger-scale GWAS summary data and a more diverse array of genetic instruments. This approach will be instrumental in validating and expanding upon our findings.

## 5 Conclusion

Our study did not find evidence for a direct causal relationship between thyroid function levels and infertility in both sexes. The findings suggest that overall thyroid function levels may not be a significant predictor of infertility risk. Future research, including additional RCTs with more detailed categorization of thyroid function status, may provide further insights into the complex relationship between thyroid function and fertility.

## Data Availability

The original contributions presented in the study are included in the article/**Supplementary Material**. Further inquiries can be directed to the corresponding authors. The two-sample MR approach is based on data freely available from the public domain.

## Supplementary material

Supplementary Figure S1 Association of genetically predicted thyroid function with risk of female infertility, analyzed with four different Mendelian randomization (MR) methods. SNPs, single-nucleotide polymorphisms; OR, odds ratio; 95% CI, 95% confidence interval.

Supplementary Figure S2 Association of genetically predicted thyroid function with risk of male infertility, analyzed with four different Mendelian randomization (MR) methods. SNPs, single-nucleotide polymorphisms; OR, odds ratio; 95% CI, 95% confidence interval.

Supplementary Figure S3 Association of genetically predicted thyroid function with risk of endometriosis related infertility, analyzed with four different Mendelian randomization (MR) methods. SNPs, single-nucleotide polymorphisms; OR, odds ratio; 95% CI, 95% confidence interval.

Supplementary Figure S4 Association of genetically predicted thyroid function with risk of cervigal, vaginal, other or unspecified origin infertility, analyzed with four different Mendelian randomization (MR) methods. SNPs, single-nucleotide polymorphisms; OR, odds ratio; 95% CI, 95% confidence interval.

Supplementary Figure S5 Association of genetically predicted thyroid function with risk of uterine origin infertility, analyzed with four different Mendelian randomization (MR) methods. SNPs, single-nucleotide polymorphisms; OR, odds ratio; 95% CI, 95% confidence interval.

Supplementary Figure S6 Association of genetically predicted thyroid function with risk of tubal origin infertility, analyzed with four different Mendelian randomization (MR) methods. SNPs, single-nucleotide polymorphisms; OR, odds ratio; 95% CI, 95% confidence interval.

Supplementary Figure S7 Association of genetically predicted thyroid function with risk of anovulation related infertility, analyzed with four different Mendelian randomization (MR) methods. SNPs, single-nucleotide polymorphisms; OR, odds ratio; 95% CI, 95% confidence interval.
