# Supplementary figures and images for: Causal association between thyroid function and the risk of infertility: a Mendelian randomization study
Source: Front Endocrinol (Lausanne). 2024 Oct 4;15:1425639. doi: 10.3389/fendo.2024.1425639 (PMC11486735; doi:10.3389/fendo.2024.1425639)

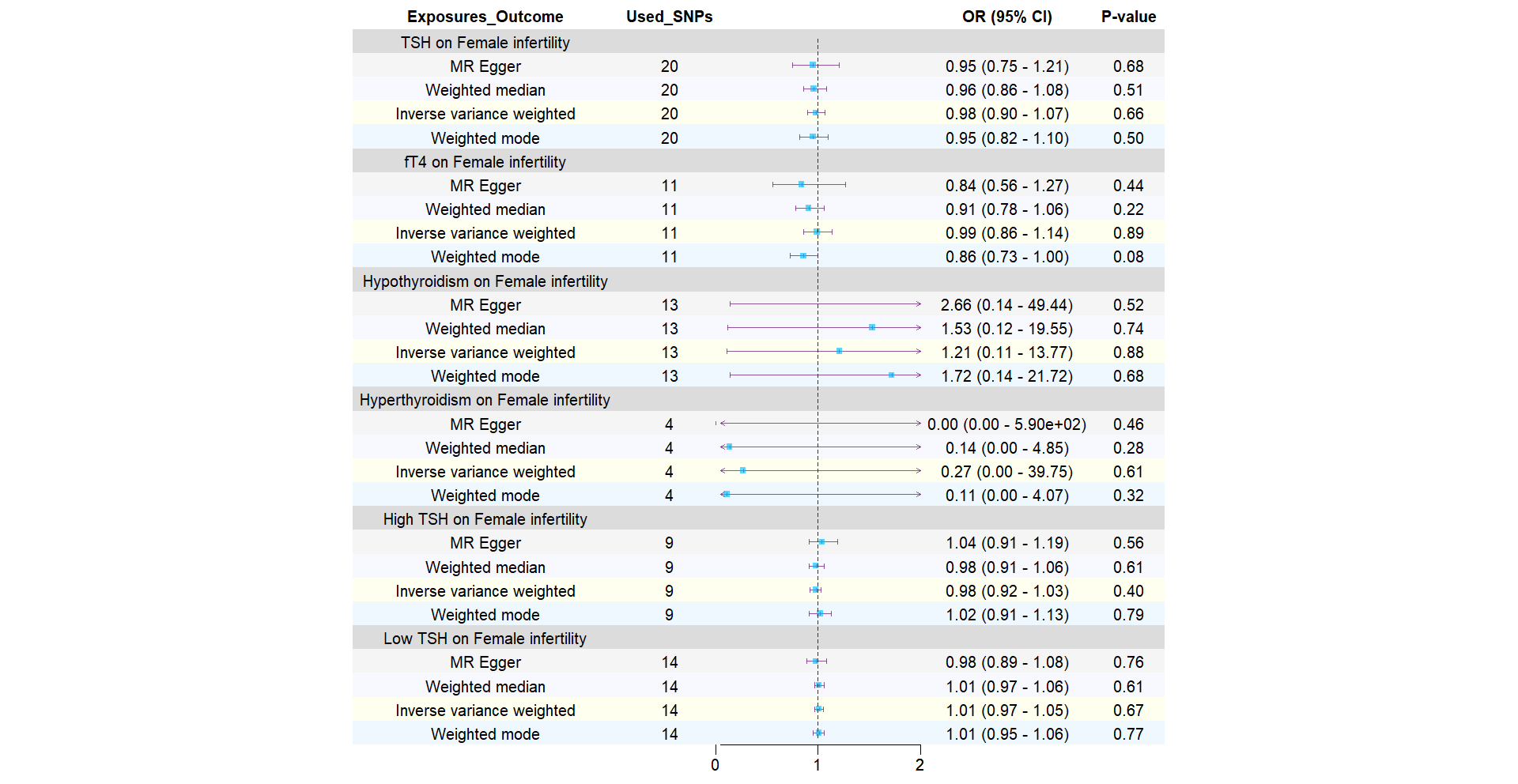

Supplement: Supplementary Figure S1 — Association of genetically predicted thyroid function with risk of female infertility, analyzed with four different Mendelian randomization (MR) methods. SNPs, single-nucleotide polymorphisms; OR, odds ratio; 95% CI, 95% confidence interval. [file Image1.jpeg]

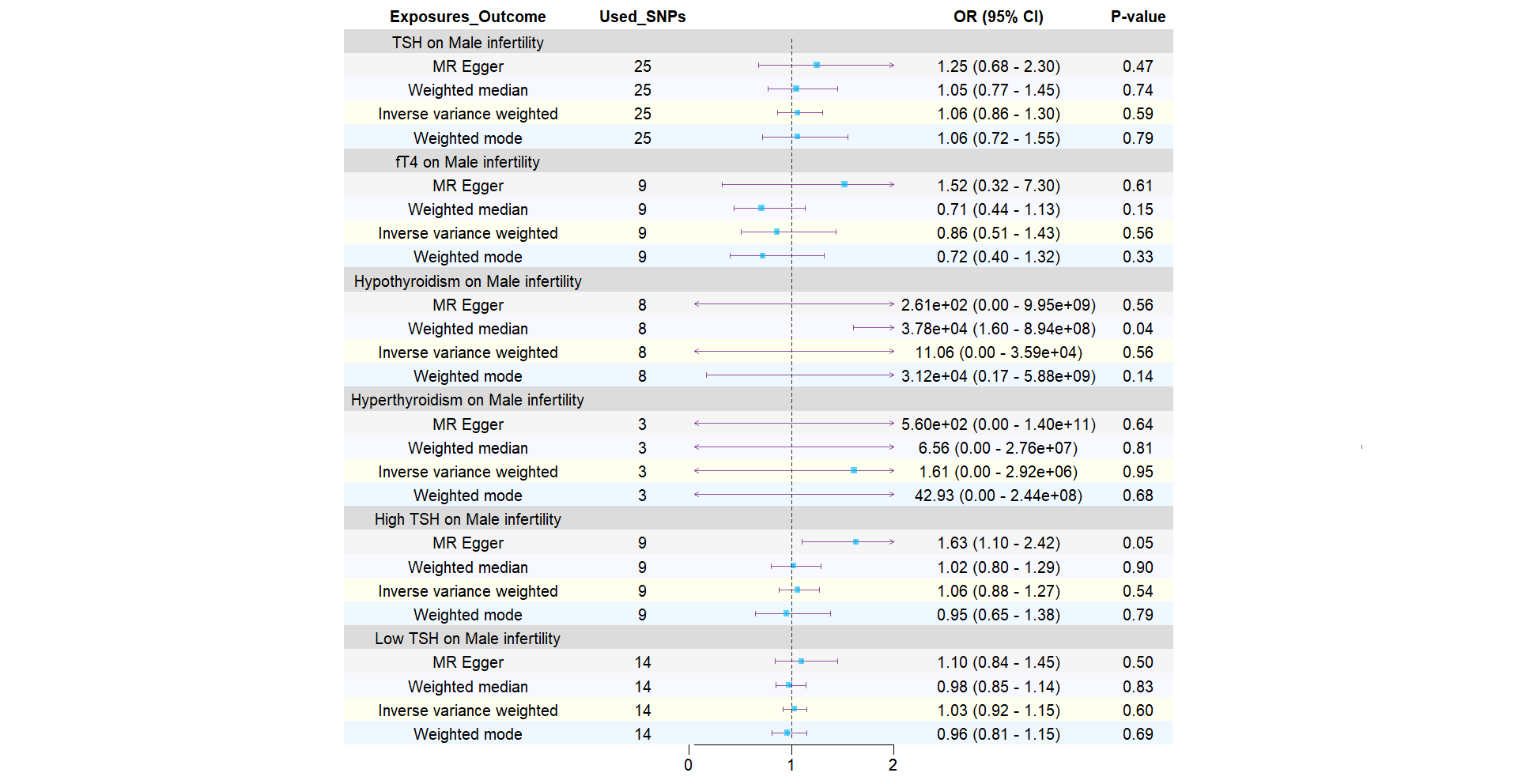

Supplement: Supplementary Figure S2 — Association of genetically predicted thyroid function with risk of male infertility, analyzed with four different Mendelian randomization (MR) methods. SNPs, single-nucleotide polymorphisms; OR, odds ratio; 95% CI, 95% confidence interval. [file Image2.jpeg]

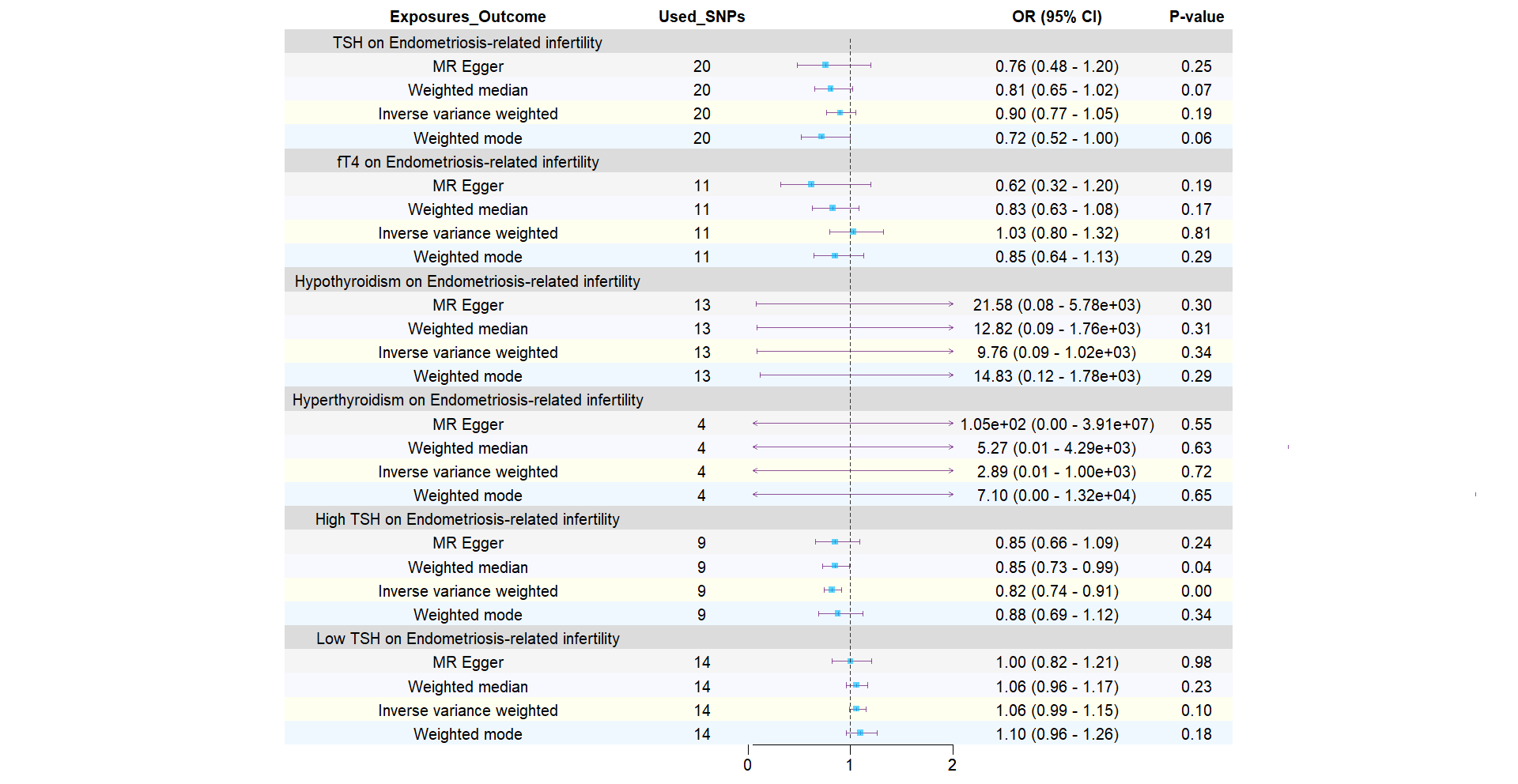

Supplement: Supplementary Figure S3 — Association of genetically predicted thyroid function with risk of endometriosis related infertility, analyzed with four different Mendelian randomization (MR) methods. SNPs, single-nucleotide polymorphisms; OR, odds ratio; 95% CI, 95% confidence interval. [file Image3.jpeg]

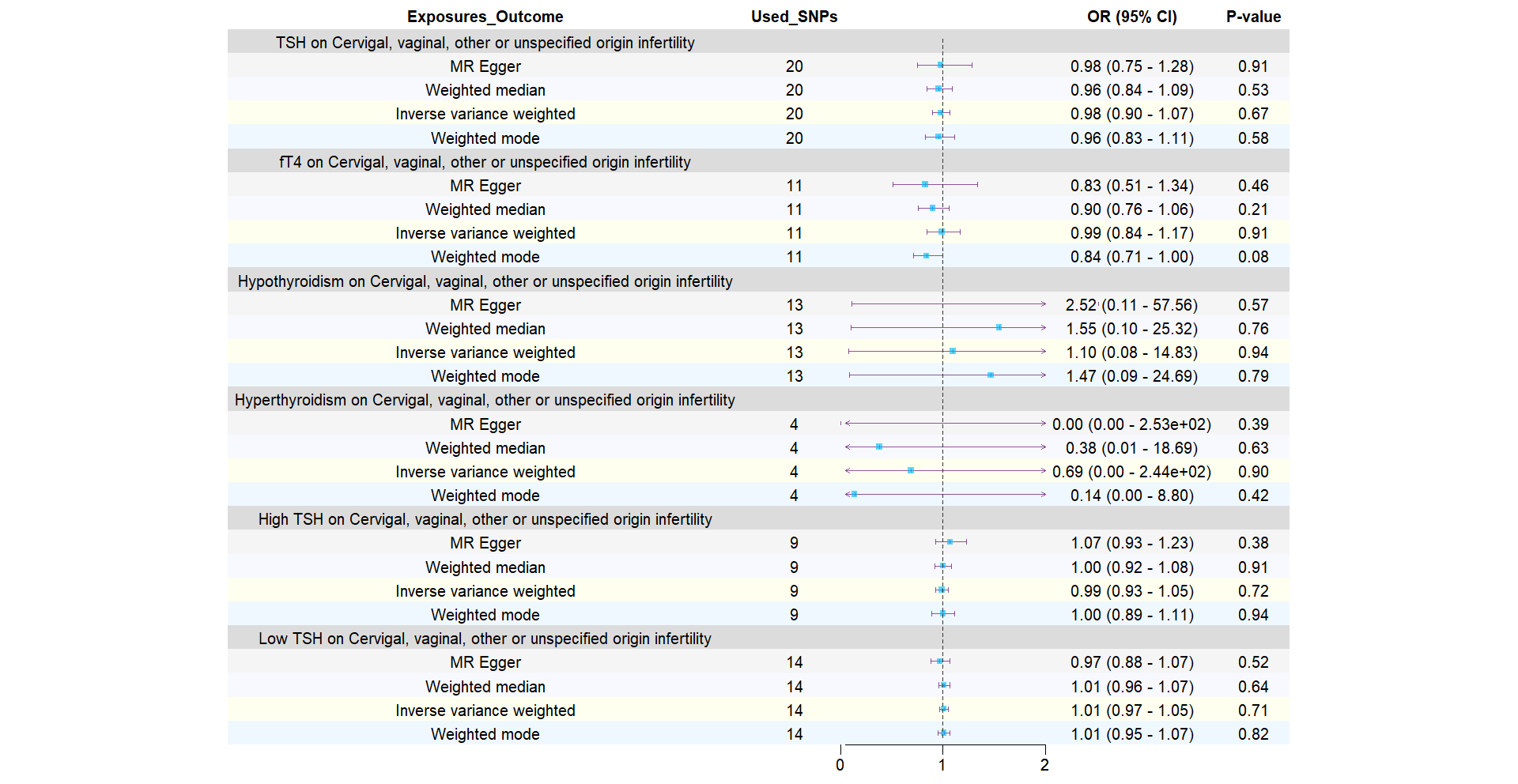

Supplement: Supplementary Figure S4 — Association of genetically predicted thyroid function with risk of cervigal, vaginal, other or unspecified origin infertility, analyzed with four different Mendelian randomization (MR) methods. SNPs, single-nucleotide polymorphisms; OR, odds ratio; 95% CI, 95% confidence interval. [file Image4.jpeg]

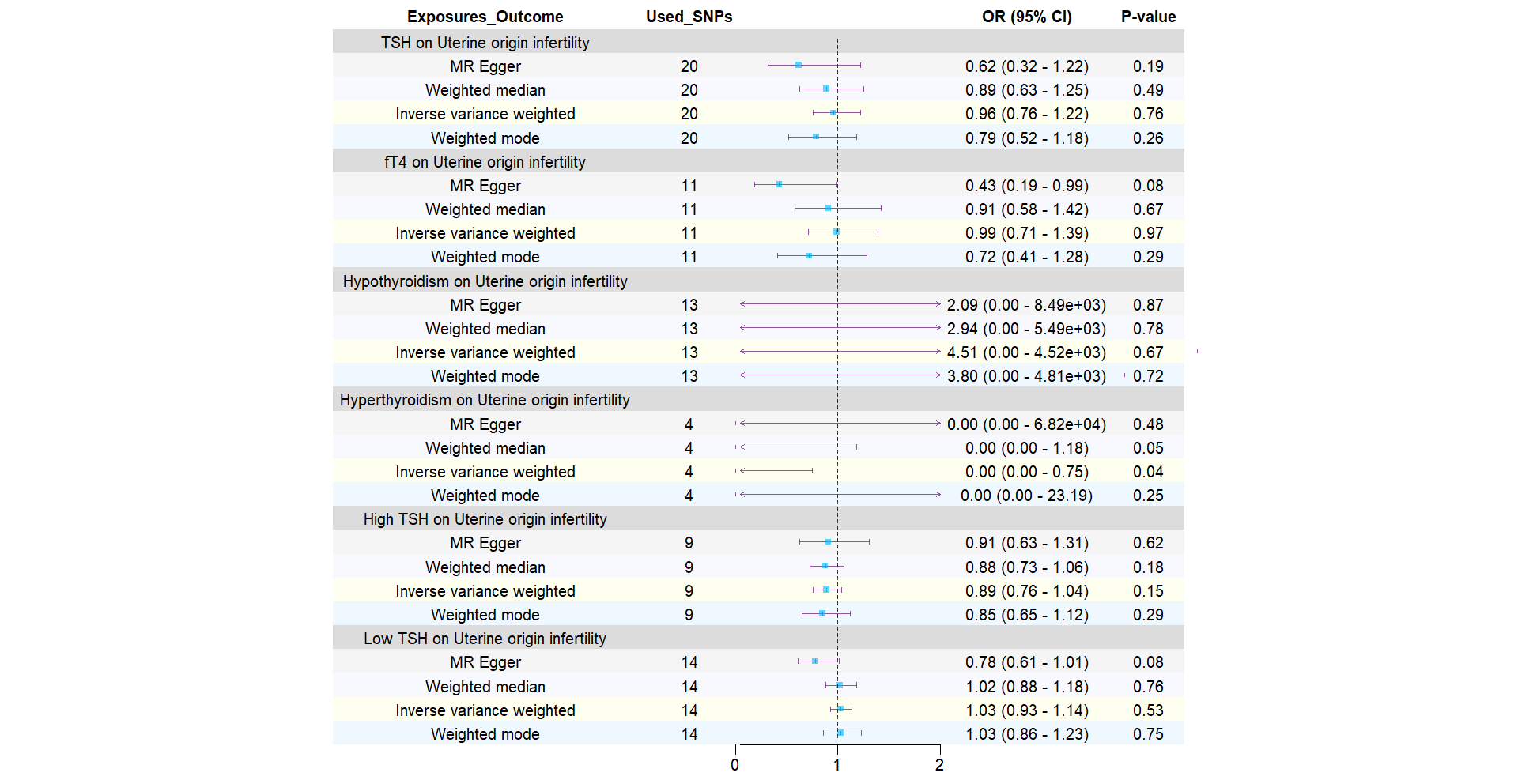

Supplement: Supplementary Figure S5 — Association of genetically predicted thyroid function with risk of uterine origin infertility, analyzed with four different Mendelian randomization (MR) methods. SNPs, single-nucleotide polymorphisms; OR, odds ratio; 95% CI, 95% confidence interval. [file Image5.jpeg]

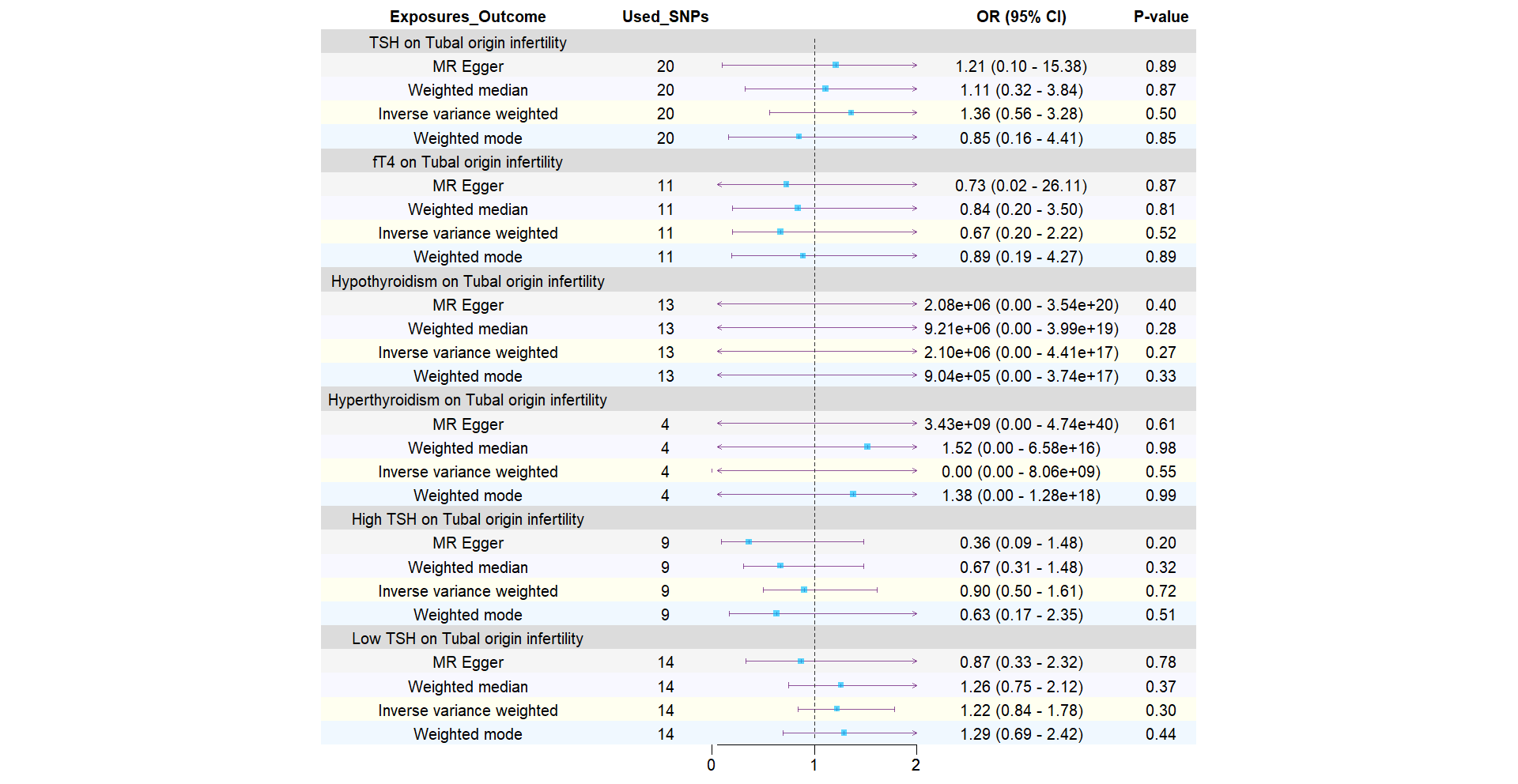

Supplement: Supplementary Figure S6 — Association of genetically predicted thyroid function with risk of tubal origin infertility, analyzed with four different Mendelian randomization (MR) methods. SNPs, single-nucleotide polymorphisms; OR, odds ratio; 95% CI, 95% confidence interval. [file Image6.jpeg]

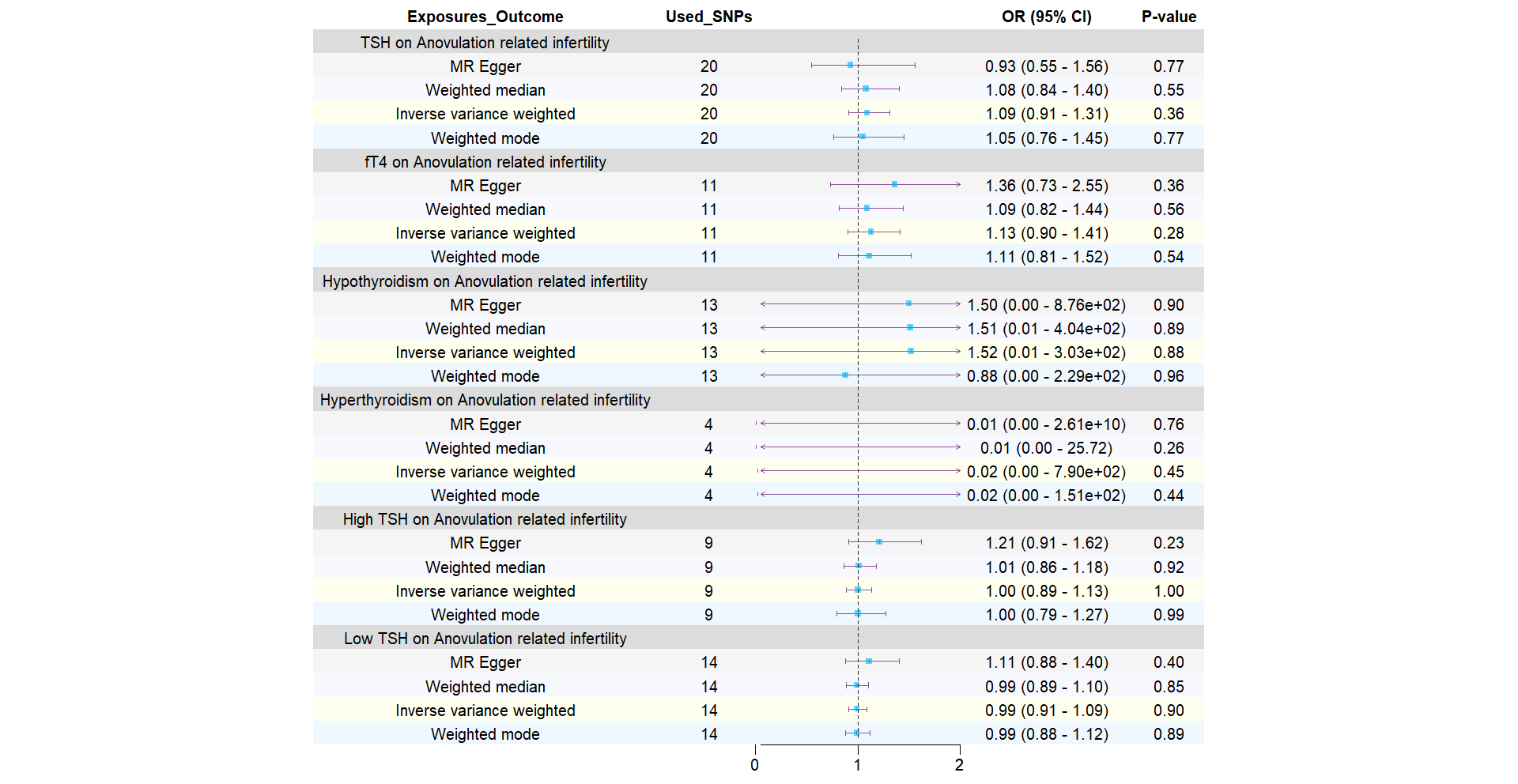

Supplement: Supplementary Figure S7 — Association of genetically predicted thyroid function with risk of anovulation related infertility, analyzed with four different Mendelian randomization (MR) methods. SNPs, single-nucleotide polymorphisms; OR, odds ratio; 95% CI, 95% confidence interval. [file Image7.jpeg]
